# Equation-Method for correcting clipping errors in OFDM signals

**DOI:** 10.1186/s40064-016-2413-0

**Published:** 2016-06-30

**Authors:** Nargis Bibi, Anthony Kleerekoper, Nazeer Muhammad, Barry Cheetham

**Affiliations:** Department of Computer Science, Fatima Jinnah Women University, The Mall, Rawalpindi, Pakistan; School of Computing, Mathematics and Digital Technologies, Manchester Metropolitan University, Manchester, UK; Department of Mathematics, Comsats Institute of Information technology, Wah Cantt, Pakistan; Department of Computer Science, The University of Manchester, Manchester, UK

**Keywords:** Clipping, Orthogonal frequency division multiplexing (OFDM), FFT, Square matrix, Equation-Method, Symbol error probability

## Abstract

Orthogonal frequency division multiplexing (OFDM) is the digital modulation technique used by 4G and many other wireless communication systems. OFDM signals have significant amplitude fluctuations resulting in high peak to average power ratios which can make an OFDM transmitter susceptible to non-linear distortion produced by its high power amplifiers (HPA). A simple and popular solution to this problem is to clip the peaks before an OFDM signal is applied to the HPA but this causes in-band distortion and introduces bit-errors at the receiver. In this paper we discuss a novel technique, which we call the Equation-Method, for correcting these errors. The Equation-Method uses the Fast Fourier Transform to create a set of simultaneous equations which, when solved, return the amplitudes of the peaks before they were clipped. We show analytically and through simulations that this method can, correct all clipping errors over a wide range of clipping thresholds. We show that numerical instability can be avoided and new techniques are needed to enable the receiver to differentiate between correctly and incorrectly received frequency-domain constellation symbols.

## Background

Orthogonal frequency division multiplexing (OFDM) is a multicarrier modulation technique which is used in LTE and WiMAX as well as some older systems such as ADSL and DAB radio (Dahlman et al. 2011; Ergen 2009). The main advantages of OFDM over conventional single carrier systems are its spectral efficiency and imperviousness to multipath effects. However, OFDM has a few disadvantages and one of the most severe is its relatively high peak to average power ratios (PAPR). This is an inevitable consequence of the use of Fast Fourier Transforms (FFT) with many frequency-domain constellation symbols in the same phase. The PAPR increases linearly with the number of subcarriers and the maximum PAPR is equal to the number of subcarriers. In modern systems there can be as many as 2,048 subcarriers leading to very severe PAPR.

The high peaks can produce serious in-band nonlinear distortion when applied to the high power amplifier (HPA) at the transmitter unless the HPA is highly linear with a large back-off and the transmitter’s digital to analogue converter also has a large dynamic range. Although these may be feasible for high-power devices such as base transceiver stations, they are too expensive in terms of power consumption for use in mobile devices which makes high PAPR a serious problem for mobile telephone and Internet services.

Significant research effort has been focused on this problem and solutions fall into roughly two groups: prevention and mitigation. Prevention methods work on the transmitter side and either aim to generate distortionless OFDM symbols or reduce PAPR but introduce other types of distortions. Examples of prevention methods include coding (Zhang et al. 1999), selected mapping (Kim et al. 2010; Heo et al. 2007), partial transmit sequences (Weng et al. 2013), tone reservation (Park and Park 2012) active constellation extension and soft-clipping and filtering (Bae et al. 2010; Ochiai and Imai 2002). Mitigation techniques work on the receiver side and aim to correct any distortions resulting either from high PAPR or from prevention techniques. Examples include Bussgang Noise Cancellation (Chen and Haimovich 2003) and Decision Aided Reconstruction (Kim and Stuber 1999). There are other methods in the literature which have been proposed after modifying or optimizing these basic techniques, such as peak insertion (Siddiq 2015; Yang et al. 2012).

One of the simplest and most popular prevention techniques is soft-clipping which reduces high peaks by artificially limiting the maximum amplitude of any time-domain sample to a pre-defined threshold before it is applied to the amplifier (Ochiai and Imai 2000). The clipping is a non-linear process which introduces bit-errors at the receiver in a hard to predict manner. This paper discusses a novel method for correcting the bit-errors introduced by soft clipping, which we call the Equation-Method. The basis of the method is the generation of a set of simultaneous equations from the Fast Fourier Transform (FFT) equations taking the clipped time-domain samples as unknowns. When the equations are solved, the amplitude of the time-domain samples, before clipping, are found thereby effectively reversing the clipping at the receiver. This paper expands on our previous work by more thoroughly analyzing the theoretical foundations of the method (Bibi et al. 2013).

The rest of this paper is organized as follows. The next section presents some related work on PAPR reduction. “The soft-clipping technique” section presents the clipping method in more detail. In “The Equation-Method” section we present our Equation-Method with mathematical and simulation analysis of an ideal scenario. “Equation-Method in practice” section investigates the application of the Equation-Method to more realistic scenarios. This is followed in “Discussion” section with a discussion of our results before we draw some conclusions and present future directions in “Conclusion” section.

## Related work

A baseband OFDM signal is expressed as:

1 $$\begin{aligned} x(n)=\frac{1}{\sqrt{N}}\sum _{k=0}^{N-1}X_{k}e^{\frac{j2\pi nk}{N}}, \end{aligned}$$

where N is the number of subcarriers and $X_k$ for $k=0,1,\ldots ,N-1$ are the complex modulated data symbols. Any cyclic extension is disregarded.

The PAPR of the OFDM symbol is defined as the ratio of the maximum to the average power of the signal. Mathematically it can be written as:

2 $$\begin{aligned} { PAPR}=\frac{max_{0\le \text {n}<{N-1}}\left| x(n)\right| ^2}{E\{\left| x(n)\right| ^2\}}, \end{aligned}$$

When a number of frequency-domain constellation points are in the same phase then high peaks can occur and these peaks are likely to fall in the non-linear range of the amplifier causing distortions and increased bit error rates (BER). Significant research efforts have been invested into addressing this problem and, as mentioned, solutions fall, roughly, into two groups: prevention and mitigation. Prevention techniques work on the transmitter side to avoid distortions caused by the amplifier whereas mitigation techniques aim to correct the errors on the receiver side.

One approach taken for prevention is to generate a number of OFDM symbols from a given bit-stream and only pass the symbol whose PAPR is below some acceptable threshold to the HPA (Jiang and Wu 2008). This method requires the addition of side information so that the receiver can decode the symbol correctly and therefore the data rate is reduced. Block coding (Zhang et al. 1999), partial transmit sequences (Weng et al. 2013) and selected mapping (Kim et al. 2010) are examples of techniques for generating symbols with low PAPR. Companding has been proposed for reducing the PAPR using a compressor at the transmitter and an expander at the receiver (Wang et al. 2013). The technique reduces the PAPR sufficiently but the BER increases at the receiver in the process of expanding. It also increases the noise in the signal. The simplest and most popular method for prevention is soft-clipping and filtering which we discuss in more detail in the next section (Ochiai and Imai 2000, 2002).

Mitigation techniques attempt to reduce the BER at the receiver. Attempts have been made to use Forward Error Correction codes but since these codes were designed for Additive White Gaussian Noise they are not effective without significant modification. Others have proposed the use of iterative receivers such as Bussgang Noise Cancellation (BNC) and Decision Aided Reconstruction (DAR) (Chen and Haimovich 2003; Kim and Stuber 1999; Bibi and Cheetham 2012). BNC makes use of Bussgang Theorem (Rowe 1982) to estimate the clipping noise at the receiver and remove it from the received signal. DAR, on the other hand, attempts to regrow the original peaks by iteratively passing the received signal through an FFT/IFFT pair. These approaches can be computationally expensive owing to their iterative nature.

An alternative mitigation technique is Active Constellation Extension which distorts the frequency-domain constellation points at the transmitter in such a way that they can be correctly received despite the distortions resulting from PAPR (Bae et al. 2010). This approach uses more power because the signal is spread wider.

In this paper we discuss a hybrid method which attempts to correct the errors introduced by the soft-clipping prevention approach.

## The soft-clipping technique

Soft-clipping is a very simple method for preventing high PAPR (Ochiai and Imai 2002). The transmitter selects a threshold amplitude, *A*, and clips all time-domain samples to that threshold before the signal is passed to the amplifier. With reference to Eq. (1), the clipping method can be expressed as:

3 $$\begin{aligned} x_c(n)={\left\{ \begin{array}{ll} x_n,&{} \quad \text {if }\;\left| x_n\right| \le A\\ A \cdot e^{j\theta \left( n\right) },&{} \quad \text {if }\; \left| x_n\right| >A \end{array}\right. } \end{aligned}$$

where $x_c(n)$ is the clipped signal.

Although soft-clipping avoids the distortions resulting from the non-linear region of the HPA, it does introduce new bit errors of its own. Nevertheless, the errors introduced by HPA are harder to correct than those caused by soft-clipping (LUO 2011). If the clipping is applied to the Nyquist sampling rate then the resultant distortion falls in-band whereas if the signal is oversampled then the distortion is distributed both in- and out-of-band and the out-of-band distortion can be removed by applying frequency-domain filtering (Armstrong 2001). However, filtering causes peak regrowth in the signal. To overcome the peak regrowth, clipping and filtering is repeated several times until the desired reduction in PAPR is achieved (Armstrong 2002).

In this paper we focus only on in-band distortion assuming the Nyquist Sampling rate and do not consider the effect of out-of-band or additive noise.

## The Equation-Method

The Equation-Method is a method of mitigating in-band distortions caused by soft-clipping. At the receiver, the time-domain signal is passed through an Nth order FFT to convert it into frequency-domain constellation symbols. This can be expressed in matrix form as:

4 $$\begin{aligned} \underline{Y}=P\cdot \underline{x} \end{aligned}$$

where $\underline{x}$ and $\underline{Y}$ are the $N \times {1}$ vectors of time-domain and frequency-domain samples respectively, and the elements of the $N \times N$ matrix *P* are as follows:

5 $$\begin{aligned} p(n,k) = exp(-2 \pi j (n-1) (k-1) / N), \end{aligned}$$

The resulting constellation symbols can be mapped onto real and imaginary axes. The valid constellation points for all modulation techniques (PSK, QPSK, 16-QAM, etc.) are pre-defined at known, fixed positions on the complex plane but since the signal has been clipped the received constellation symbols may not be at one of those fixed positions. The receiver therefore moves each symbol to the nearest known valid point based on the Euclidean distance between the received symbol location and the points. We refer to this process as “snapping”. The distortions produced by soft-clipping can result in bit errors if the snapping process incorrectly places a received symbol on an incorrect point.

By the nature of the FFT, each constellation symbol can be considered a linear sum of every time-domain amplitude where the amplitudes are the pre-clipped amplitudes. This makes it possible to recover the pre-clipped amplitudes from correctly received constellations and thereby correctly snap every constellation and remove all bit-errors introduced by soft-clipping.

If the receiver knows the clipping amplitude, *A*, (which is a reasonable assumption) then it can easily identify which time-domain samples have been (or potentially have been) clipped and can therefore divide the received time-domain samples into clipped samples, $x_c$ and unclipped samples, $x_u$. The amplitudes of the unclipped samples can be considered reliable because they have not been affected by soft-clipping. On the other hand, the amplitudes of the clipped samples are unreliable and we treat them as unknown variables in a modified FFT equation.

As we have mentioned, some of the snapped constellation symbols will be correct and some will be incorrectly snapped. We can therefore divide the received, frequency-domain constellation symbols also into two groups: $Y_R$ as the reliable (correctly snapped) symbols and $Y_U$ as the unreliable (incorrectly snapped) symbols. Dividing the received complex time-domain samples *x*(*n*) for $n=1,2,\ldots ,N,$ into clipped samples ${x_c(n)}$ for $n=1,\ldots ,L$ and non-clipped samples ${x_u(n)}$ for $n=1,\ldots ,N-L$ allows the initial FFT Eq. (4) at the receiver to be partitioned as:

6 $$\begin{aligned} \left[ \begin{array}{c} \underline{Y}_R\\ \underline{Y}_U\\ \end{array}\right] = \begin{bmatrix} G&\quad F \\ E&\quad D \\ \end{bmatrix} \times \begin{bmatrix} \underline{x}_c\\ \underline{x}_u\\ \end{bmatrix}, \end{aligned}$$

where matrices *D*, *E*, *F* and *G* are the complex exponentials from matrix *P* in Eq. (4) for the relevant FFT equations. The matrix *P* is partitioned such that *G* and *E* are matrices of the sinusoidal coefficients associated with the time-domain samples $\underline{x}_c$, whereas *F* and *D* are matrices of the sinusoidal coefficients associated with the $\underline{x}_u$ respectively. Furthermore, the sub-matrices *G* and *F* correspond to the reliable frequency-domain symbols $\underline{Y}_R$, whereas *E* and *D* correspond to the unreliable frequency domain symbols $\underline{Y}_U$. The dimensions of $\left( \underline{{Y}}_R\right)$ and $(\underline{{Y}}_U)$ are defined as $M\times 1$ and $(N - M) \times 1$ respectively. The dimensions of $\left( \underline{{x}}_c\right)$ and $(\underline{{x}}_u)$ are defined as $L\times 1$ and $(N - L) \times 1$ respectively. We can find the true values of the clipped time-domain samples, $\underline{x_c}$, by treating them as unknowns. Since each constellation symbol can be written as the sum of every time-domain sample, we can write:

7 $$\begin{aligned} \underline{Y}_R = G \underline{x}_c + F \underline{x}_u. \end{aligned}$$

When $L=M$,and *G* is non-singular, rearranging the equation shows how the true, time-domain samples of the clipped samples can be found from the reliable constellation points.

8 $$\begin{aligned} \underline{{x}}_c = G^{-1} (\underline{{Y}}_R - F \underline{x}_u) \end{aligned}$$

Assuming that there are *L* clipped samples then we need to find *L* reliable constellation symbols to generate the set of equations to provide one equation per unknown. Unfortunately, the receiver cannot know for certain which snapped constellation symbols are reliable (correctly snapped) and which are not. However, there are usually more correctly snapped constellations than incorrectly snapped and also many more correctly snapped constellations than clipped samples.

To show this we simulated 10,000 randomly generated OFDM symbols with 64 subcarriers, each modulated with 16-QAM, using a MATLAB simulation. The clipping ratio is defined as the ratio of the clipping threshold *A* to the root mean square (RMS) value $\sigma$ of the OFDM signal. The clipping threshold was varied from 0.4 to 0.7 which correspond to a clipping ratio of between approximately 1.01 and 1.79 since the average power of the OFDM signal was 0.156 over the 10,000 symbols. The range of clipping thresholds therefore encompasses mild to very severe clipping. For each clipping threshold we recorded the average number of correctly snapped constellation symbols and the number of clipped time-domain samples.

**Fig. 1:**
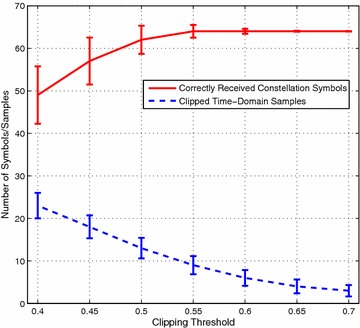
The average number of correctly snapped constellation symbols and clipped time-domain samples

The results in Fig. 1 show that even with severe clipping there are more reliable than unreliable constellation symbols and many more than clipped samples. Since we only need one reliable constellation per clipped sample, the Equation-Method is a feasible approach.

As a proof of concept we simulated 10, 000 OFDM symbols following the same process as above and applied the Equation-Method to correct the clipping errors, assuming that the receiver is aware of which constellation symbols are correctly snapped. We found that at all clipping threshold, the Equation-Method corrected $100\,\%$ of the errors except at the most severe clipping threshold $(\hbox {A}=0.4)$ where 2 of the 10,000 OFDM symbols could not be corrected. Those two cases were because there were more clipped samples than correctly snapped constellation symbols.

These results prove that the Equation-Method can correct all bit errors introduced by soft clipping. However, this assumes that the receiver is aware of which constellation symbols are correctly snapped which, in practice, it cannot know. In the next section we apply the Equation-Method in a realistic scenario where the receiver does not know which constellation symbols are reliable.

## Equation-Method in practice

In the previous section we have shown that the Equation-Method can correct all clipping errors as long as it correctly selects reliable symbols. In this section we investigate the application of the Equation-Method when the receiver does not know which constellation symbols are reliable.

**Fig. 2:**
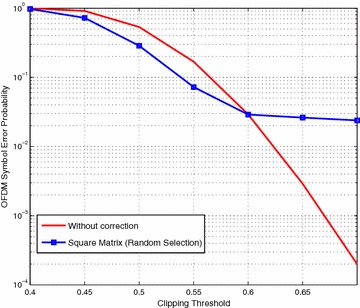
OFDM symbol error probability using Equation-Method with square matrix in real figure legend text

Initially, we consider a strategy whereby the receiver randomly selects constellation symbols for use in Eq. (8). Following the same procedure as described in the previous section we apply the Equation-Method using this random selection. We found the OFDM symbol error probability which is the probability that the OFDM symbol contains no bit errors which is a more useful measure in practical situations and sets a higher standard since all bit errors in a symbols must be corrected. The results in Fig. 2 show that the method reduces the symbol error probability at severe to moderate clipping thresholds but has worse performance at the mild clipping thresholds.

On closer examination we see that the symbol error probability using this method cannot drop below approximately 3 % and this is because of the existence of singular matrices. In order to solve Eq. (8) it must be possible to invert matrix *G* but if *G* is singular then inverting it produces incorrect solutions to the equations and hence the symbol is in error.

**Fig. 3:**
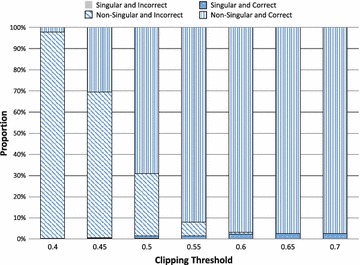
The nature of the matrix *G* in terms of singularity and correct symbols

Following the same procedure we simulated 250 OFDM symbols and for each one created 250 matrices by randomly selecting constellation symbols. We tested each matrix to determine whether the matrix was singular and/or whether it contained any incorrect symbols. The results in Fig. 3 show that with severe clipping the majority of matrices are non-singular but contain incorrect symbols. As the clipping threshold increases towards mild clipping, almost all of the matrices are non-singular with only correct symbols. However, as the clipping threshold increases there becomes a larger proportion of matrices that are singular, up to around $3\,\%$. This is because at more moderate thresholds, the matrices become smaller which increases the probability of them being singular.

The results show that to continue to achieve improved results at mild clipping thresholds, singular matrices must be avoided. Fortunately, it is relatively trivial to test for singularity and we therefore consider a modification to the Equation-Method whereby the randomly selected set of constellation symbols is discarded if the resulting matrix *G* is singular. In that case a new set is randomly selected and tested until a set is found with a non-singular matrix. We place a limit on the maximum number of iterations at 50 though in practice we never needed more than a few iterations.

**Fig. 4:**
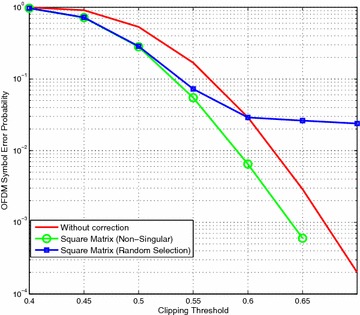
Equation-Method with non-singular matrices

Following the same experimental procedure with 10,000 OFDM symbols we applied this modified version of the Equation-Method. The results in Fig. 4 show that avoiding the singular matrices allows the bit error correction to continue at mild clipping thresholds.

## Discussion

The results in the previous sections show that the Equation-Method is feasible and is able to correct bit errors and reduce the symbol error probability. However, we have seen that the method faces two problems. Firstly, if the matrix *G* is singular then the equations will have no solution and the OFDM symbol will be in error. We were able to avoid this problem.

The second problem is that we may select incorrectly snapped constellation symbols for use in our equations. If this happens then the solution found will be incorrect and bit errors will not be corrected. This is a more severe problem as the probability of including an incorrectly snapped constellation is usually higher than of selecting a singular matrix; it only requires one incorrect constellation symbol to produce incorrect solutions and there is no method to determine with certainty which symbols are correctly or incorrectly snapped.

In our earlier work we avoided the issue of singularity and mitigated the problem of incorrect symbols by using a pseudo-inverse approach. In this approach we do not select one equation per unknown but instead utilize all the constellation symbols to create a thin-rectangular matrix *G* which can be inverted through the Penrose–Moore pseudo-inverse function (Penrose 1956). The pseudo-inverse will provide a solution even for badly scaled matrices. Moreover, if there are many more correctly snapped constellations than incorrectly snapped then the impact of incorrectly snapped is not noticed because the correctly snapped constellations dominate the solution.

**Fig. 5:**
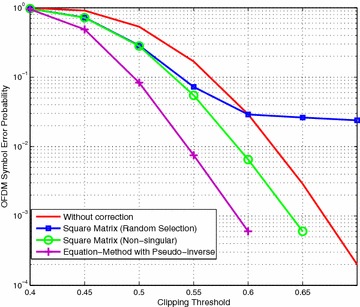
Comparison of Equation-Method using pseudo-inverse and square matrix solutions

Figure 5 shows that the pseudo-inverse method produces better results than using the square matrix method because it mitigates the impact of incorrectly snapped constellations. However, in our earlier work we did not consider why the pseudo-inverse might be more effective than using a square matrix and we have addressed that in this paper.

Despite previously published results (Bibi et al. 2013) showing that the pseudo-inverse approach performs well, we still believe that the square matrix approach has the potential for even better performance. The square matrix approach selects fewer constellation symbols and therefore has a lower chance of selecting an incorrect equation. On the other hand, the consequence of selecting an incorrect equation is more serious for the square matrix approach than for the pseudo-inverse approach. In our earlier work (Bibi et al. 2013) we considered some techniques for reducing the probability of selecting incorrect symbols and applied those techniques to the pseudo-inverse approach. If those methods were applied to the square-matrix approach it may outperform the pseudo-inverse method by selecting only correct constellations.

In some cases, it is possible to detect that a solution produced by the square matrix approach is incorrect. For example, if the recalculated amplitudes of the clipped time-domain samples are found to be less than the clipping threshold, they are clearly not correct. The selection of constellations can then be modified.

## Conclusion

This paper has investigated the correction of bit-errors caused by clipping distortion in OFDM symbols. We have investigated the application of the Equation-Method using one equation per unknown showing that the problem of including incorrectly snapped constellation symbols in the set of reliable constellations restricts its effectiveness. Nevertheless, we have proved analytically and through simulation that the Equation-Method is capable of correcting all bit errors except at the most severe clipping threshold when there are more clipped samples than reliable constellations.

Our work opens up a new approach to dealing with high PAPR through clipping and the correction of clipping errors. New techniques are required to aid in differentiating reliably from unreliably snapped constellations to improve the performance. We have briefly considered some of these methods in the context of a pseudo-inverse approach to the Equation-Method and will consider them in the context of the square-matrix approach in our future work. Performance of the square-matrix in the presence of additive white Gaussian noise will also be addressed in the future work.
